# Supplementary material for: Experimental Evidence for Double Quaternary Azeotropy’s Existence
Source: Entropy (Basel). 2023 Jun 26;25(7):980. doi: 10.3390/e25070980 (PMC10377813; doi:10.3390/e25070980)
Supplement: Supplementary file 1 [file entropy-25-00980-s001.zip › entropy-2451540-supplementary.pdf]

The Supplementary Materials

Table S1: The comparison of experimental and calculated azeotrope compositions at 101.3 kPa (for different thermodynamic models).

Azeotrope	Calculated data					
	UNIFAC		UNIF-LL		NRTL-HOC	
	$X_1(X_2)$, mole frac.	u_r	$X_1(X_2)$, mole frac.	u_r	$X_1(X_2)$, mole frac.	u_r
ACN + W	0.6692	0.023	0.6871	0.008	0.6709	0.014
ACN + CHEN	0.4929	-	0.4726	-	0.4877	-
CHEN + W	0.6810	0.012	0.6849	0.009	0.6815	0.014
CHL + W	0.8373	0.003	0.8371	0.003	0.8373	0.003
ACN + W + CHEN	0.3566 (0.1992)	-	0.3203 (0.1994)	-	0.3709 (0.1866)	-
ACN + CHEN + CHL	0.3728 (0.3717)	-	0.3811 (0.3007)	-	0.4850 (0.5088)	-
ACN + W + CHEN + CHL	0.341 (0.1889 / 0.4009)	-	0.2686 (0.2105 / 0.3211)	-	No Azeotrope	

Table S2: The comparison of experimental and calculated azeotrope boiling point at 101.3 kPa (for different thermodynamic models).

Azeotrope	Calculated data					
	UNIFAC		UNIF-LL		NRTL-HOC	
	T, K	u_r	T, K	u_r	T, K	u_r
ACN + W	348.60	0.001	351.18	0.024	350.08	0.010
ACN + CHEN	338.29	0.019	339.43	0.002	339.79	0.002
CHEN + W	343.93	0.003	343.65	0.004	373.95	0.000
CHL + W	329.00	0.004	329.02	0.004	329.14	0.002
ACN + W + CHEN	335.06	0.005	335.33	0.016	334.34	0.001
ACN + CHEN + CHL	339.08	-	341.91	-	339.79	-
ACN + W + CHEN + CHL	334.02	-	336.09	-	No Azeotrope	

Table S3: The comparison of experimental and calculated LLE in binary constituents at 293.15 K (for different thermodynamic models).

ACN + CHEN		W + CHEN		W + CHL	
X_1', mole frac. (u_r)	X_2'', mole frac. (u_r)	X_1', mole frac. (u_r)	X_2'', mole frac. (u_r)	X_1', mole frac. (u_r)	X_2'', mole frac. (u_r)
**Experimental data**					

**0.7610**	**0.8210**	**0.9999**	**0.9981**	**0.9989**	**0.9908**
Calculated data by NRTL					
0.7686	0.8649	0.9999	0.9985	0.9986	0.9958
(0.01)	(0.05)	(0.00)	(0.0004)	(0.0003)	(0.005)
Calculated data by UNIFAC					
0.7529	0.8532	0.9998	0.9868	0.9989	0.9954
(0.01)	(0.04)	(0.0001)	(0.011)	(0.017)	(0.005)
Calculated data by UNIF-LL					
0.5993	0.8363	0.9996	0.9987	0.9989	0.9946
(0.21)	(0.019)	(0.0003)	(0.0006)	(0.000)	(0.004)
Calculated data by NRTL-HOC					
0.6197	0.8191	0.9999	0.9978	0.9982	0.9938
(0.18)	(0.003)	(0.000)	(0.0003)	(0.007)	(0.003)

Table S4: The comparison of experimental and calculated LLLE in ternary constituents at 293.15 K (for different thermodynamic models).

ACN liquid phase		CHEN liquid phase		W liquid phase	
X_W, mole frac.	X_{ACN}, mole frac.	X_W, mole frac.	X_{ACN}, mole frac.	X_W, mole frac.	X_{ACN}, mole frac.
**Experimental data**					
**0.321**	**0.620**	**0.009**	**0.116**	**0.894**	**0.103**
Calculated data by NRTL					
0.258	0.673	0.004	0.094	0.890	0.109
Calculated data by UNIFAC					
0.159	0.692	0.022	0.125	0.878	0.121
Calculated data by UNIF-LL					
0.280	0.598	0.008	0.096	0.847	0.150
Calculated data by NRTL-HOC					
0.313	0.678	0.003	0.110	0.814	0.185

The analysis of the data in Tables S1-S4 shows that all models reproduced the splitting diagram structure at a qualitative level. If we compare the quantitative reproduction of the LLE and LLLE, then the preference should be given to the parameters of the NRTL model (according to the totality of the described characteristics). The UNIF-LL and NRTL-HOC models poorly reproduced solubility in the ACN + CHEN system. Significant differences were observed in the description of VLE equilibria, despite the fact that the known experimental azeotropic characteristics were reproduced with a relative standard uncertainty not exceeding 0.03. The qualitative structure of the scan of phase diagram was identical in all cases. The internal azeotrope with an intermediate boiling point was modeled by all models except for NRTL-HOC; the internal structure of composition tetrahedron differed significantly. The types and Poincaré indexes of singular points of the phase diagram obtained by calculation of VLE with UNIF-LL and NRTL-HOC are given in Tables S5 and S6.

Table S5. The types and Poincaré indexes of singular points of the phase diagram of acetonitrile (ACN) + cyclohexene (CHEN) + water (W) + chloroform (CHL) (by UNIS-LL model).

Singular Point	ACN	CHEN	W	CHL	CHL + W	ACN + CHEN	ACN + W
Type	N^{st}	N^{st}	N^{st}	S	N^{unst}	S	S
Poincare index	+1	+1	+1	0	–1	0	–1

Singular Point	CHEN + W	ACN + CHL + CHEN	ACN + CHEN + W	ACN + CHEN + W + CHL
Type	S	S	N^{unst}	S
Poincaré index	–1	–1	–1	+1
			**The sum of Poincaré indexes is 0**	

N^{st} – stable node; N^{unst} – unstable node; S – saddle

Table S6. The types and Poincaré indexes of singular points of the phase diagram of acetonitrile (ACN) + cyclohexene (CHEN) + water (W) + chloroform (CHL) (by NRTL-HOC model).

Singular Point	ACN	CHEN	W	CHL	CHL + W	ACN + CHEN	ACN + W
Type	N^{st}	N^{st}	N^{st}	S	N^{unst}	S	S
Poincaré index	+1	+1	+1	0	–1	0	–1
Singular Point	CHEN + W	ACN + CHL + CHEN	ACN + CHEN + W				
Type	S	S	S				
Poincaré index	–1	–1	+1				
			**The sum of Poincaré indexes is 0**				

N^{st} – stable node; N^{unst} – unstable node; S – saddle
